# Validity assessment of the single-point insulin sensitivity estimator (spise) for diagnosis of cardiometabolic risk in post-pubertal hispanic adolescents

**DOI:** 10.1038/s41598-020-71074-y

**Published:** 2020-09-01

**Authors:** Paulina Correa-Burrows, Estela Blanco, Sheila Gahagan, Raquel Burrows

**Affiliations:** 1 Instituto de Nutrición y Tecnología de Alimentos, Universidad de Chile (UCH), Avda. El Líbano 5524, Macul, 7830490 Santiago de Chile, Chile; 2 Child Development and Community Health, University of California-San Diego (UCSD), La Jolla, USA

**Keywords:** Endocrinology, Diagnostic markers, Paediatric research, Translational research

## Abstract

Insulin measurements are not advised for cardiometabolic risk screening in large groups. Here we assessed the accuracy of the single-point insulin sensitivity estimator (SPISE) to diagnose cardiometabolic risk in Chilean adolescents. In 678 post-pubertal adolescents (52% males, M(SD) age = 16.8 (0.2) years), height, weight, waist circumference, blood lipids, glucose, insulin, and blood pressure were measured. BMI, HOMA-IR, and SPISE were estimated; HOMA-IR values ≥ 2.6 were considered insulin resistance (IR). Metabolic syndrome (MetS) was defined with the joint IDF/AHA/NHBLI standard. Using receiver operating characteristic curves, we obtained optimal SPISE cutpoints for IR and MetS diagnosis. The prevalence of MetS and IR was 8.2% and 17.1%, respectively. In males, the optimal cutoff for MetS diagnosis was 5.0 (sensitivity: 97%; specificity: 82%), and the optimal cutoff for IR diagnosis was 5.9 (sensitivity: 71%; specificity: 83%). In females, a SPISE of 6.0 had the highest sensitivity (90%) and specificity (74%) for MetS diagnosis. A SPISE of 6.4 was the optimal cutoff for IR diagnosis; however, sensitivity and specificity were 61% and 75%. In males and female post-pubertal adolescents, SPISE had a very good and good diagnostic performance, respectively, in predicting MetS. It was an accurate diagnostic tool for IR prediction in males, but not necessarily in females.

## Introduction

Insulin resistance (IR), reduced responsiveness of a target cell or a whole organism to the insulin concentration to which it is exposed, is the prelude of major cardiometabolic disorders, such as coronary heart disease, stroke, Metabolic Syndrome (MetS), non-alcoholic fatty liver disease (NAFLD) and type-2 diabetes (T2D)^1,2^. IR may be due to several causes, including the excess of adipose tissue, especially visceral adiposity. In children and adolescents, IR has grown dramatically, closely linked to the obesity epidemic, and the spread of Western-type dietary habits and sedentary behaviors^2,3^. In Chile, one in four adolescents between 15 and 19 years of age have obesity, and 14% have MetS^4^, which suggests a rising number of individuals exposed to an early onset of serious and economically burdensome chronic illnesses. Early recognition of insulin-resistant youths may be beneficial for both clinical practice and population-based health promotion efforts.

The reference standard to evaluate IR is the hyperinsulinemic-euglycemic clamp; however, it is invasive, costly, and difficult to perform in clinical and epidemiological settings ^1^. Several surrogate biomarkers have been proposed based on fasting measurements of glucose and/or insulin: HOMA-IR, QUICKI, 1/HOMA, log(HOMA), and 1/insulin^1–3^. Pulsatility of insulin release, a relatively short half-life (~ 4–6 min), the fact that insulin assays are poorly standardized and may provide different results on the same sample, and difficulties in handling and storage can all cause problems in insulin determination and interpretation of results^5–7^. Thus, insulin measurements are not advised for the screening of IR in large groups or preventive purposes^5^. The Single-Point Insulin Sensitivity Estimator (SPISE) is a recently proposed biomarker of insulin sensitivity based on BMI, triglycerides (TG), and high-density lipoprotein cholesterol (HDL-chol). Paulmichl et al. found that SPISE was comparable to the Matsuda Index in assessments of IR in Caucasian adolescents with obesity and adults, and, performed as well as HOMA-IR and QUICKI, suggesting it was well suited as a surrogate of insulin sensitivity in these groups^8^. Most population-based health surveys have BMI values and routine lipid measurements available, which provides the opportunity to assess insulin sensitivity at the population level.

With the rising prevalence of obesity among younger age populations, the need for early screening and management of IR-related cardiometabolic risk becomes urgent, mainly when the road back to optimal blood sugar control is still possible without medication. This may help to avoid the premature emergence of hyperinsulinemia, inflammation, atherogenic dyslipidemia, and endothelial dysfunction, which are responsible for the significantly increased cardiovascular risk in individuals with impaired glucose metabolism. Here, we aimed to assess the accuracy of SPISE to diagnose cardiometabolic risk in male and female Chilean post-pubertal adolescents of all weight statuses. Also, we determined the optimal cutoff point for the diagnosis of insulin resistance (IR) and MetS in this population.

## Methods

### Study design and population

This is a cross-sectional validation study for a diagnostic test conducted in 678 16–17-year-old post-pubertal adolescents, 52% males, of low-to-middle socioeconomic status (SES). Participants are from the Santiago Longitudinal Study and were enrolled at four months of age in 1992–1996 to participate in research related to nutrition and growth as infants with follow-up at 1, 5, 10, and 16y^9^. To be eligible, they had to be full-term singletons babies delivered normally following spontaneous labor, weighing ≥ 3 kg at birth, and free of acute or chronic health problems. Participants were born during a dramatic nutritional transition from parents and/or grandparents who were exposed to child undernutrition. At 16y, they were assessed for the presence of cardiometabolic risk factors^10,11^.

### Ethical approval

Ethical approval was obtained by the IRBs of the University of Michigan, Institute of Nutrition and Food Technology (University of Chile), and the University of California, San Diego. Informed and written consent from both the participants and their primary caregivers was provided according to the norms for Human Experimentation, Code of Ethics of the World Medical Association (Declaration of Helsinki, 1995).

## Measurements

### Anthropometric and pubertal assessment at 16y

Using standardized methods, research physicians measured height (cm) to the nearest 0.1 cm, using a Holtain stadiometer, and weight (kg) to the nearest 0.1 kg, using a scale (Seca 703, Seca GmbH & Co. Hamburg, Germany). Waist circumference (WC) was measured with a non-elastic flexible tape and recorded to 0.1 cm (Seca 201, Seca GmbH & Co. Hamburg, Germany). Measurements were taken twice, with a new measurement if the difference between the first two exceeded 0.5 cm for height, 0.3 kg for weight, and 1.0 cm for WC. BMI and BMI-for-age-and-sex (BMIz) were calculated, and weight status was assessed with the 2007 World Health Organization standard^12^. Pubertal development was evaluated from a physical examination of the adolescent, using the Marshall and Tanner criteria for breast and genital stage in females and males, respectively^13,14^. At 16.8y, all participants were in Tanner stage V, denoting full sexual maturity.

### Additional cardiometabolic risk assessment at 16y

After 15 min at rest, arterial blood pressure was measured on the non-dominant arm using a standard mercury sphygmomanometer; three measurements were taken, and the mean was used for analyses. Serum total glucose, insulin, total cholesterol (TChol), TG) HDL-chol, high-sensitivity C-reactive protein (hs-CRP), and adiponectin were measured after 8–12 h overnight fast. hs-CRP was measured with a sensitive latex-based immunoassay. To avoid abnormally high hs-CRP levels, adolescents with hs-CRP of > 9.0 were excluded from the analysis when inflammation was the outcome (n = 18 or 2.6%). Radioimmunoassay (Diagnostic Products Corporation, Los Angeles, CA) was used for insulin determination, and glucose was measured with an enzymatic colorimetric test (QCA S.A., Amposta, Spain). Cholesterol profile was determined by dry analytical methodology (Vitros, Ortho Clinical Diagnostics Inc., Raritan, NJ). TG/HDL-chol ratio was calculated after dividing absolute TG levels by absolute HDL-chol levels, and LDL was estimated using the Friedewald equation: [TChol—(HDL-chol + TG/5)]. Total adiponectin was determined with the Quantikine Human Total Adiponectin Immunoassay, a 4.5-h solid-phase ELISA with a minimum detectable concentration ranging from 0.079 to 0.891 ng/mL. The homeostatic model assessment (HOMA) was used to measure insulin sensitivity [Wallace 2004]. HOMA-insulin resistance (IR) was estimated as the product of fasting glucose (mmol/l) and insulin (μU/ml) divided by the constant 22.5, with values ≥ 2.6 denoting IR^15^. SPISE was computed as follows: [600 * HDL^0.185/(TG^0.2 * BMI^1.338)]^8^. A continuous value was obtained, ranging 2.5–14.5 with higher values denoting higher insulin sensitivity. Because our participants were post-pubertal adolescents, MetS was diagnosed based on the 2009 AHA/NHLBI/IDF Joint Interim Statement^16^, for individuals 16y and older.

### Data analysis

Data were analyzed using Stata for Windows V.15.0 (Lakeway Drive College Station, Texas, USA). In all variables, the Shapiro–Wilk test was used to assess the normality assumption. Statistical analysis included Student’s *t*-test for independent data and Wilcoxon’s rank-sum test for comparison of mean or median values of anthropometric and cardiometabolic variables. The χ^2^ test was used for comparison of categorical variables. Receiver operating characteristic (ROC) analysis was used to find the optimal cutoff of SPISE for MetS and IR diagnosis in males and females. Sensitivity, specificity, likelihood ratio (LR), and area under the ROC curve (AUC) were estimated. To determine the optimal cutoffs for MetS diagnosis, the Youden Index [*J* = sensitivity-(1-specificity)] was calculated. Next, the values were verified with the likelihood ratio for a positive result (LR +). The post-test probability (the proportion of participants below cutoffs who truly have the MetS) was estimated. The DeLong’s method for pair design tested the statistical significance of the difference between the AUC, to compare the diagnostic performance of SPISE, HOMA-IR and the TG/HDL-C ratio in the prediction of MetS. Last, we checked whether our SPISE cutpoints for MetS and IR diagnosis were related to higher biological risk in the group having a SPISE below those cutpoints. Cohen’s *d* and Cliff’s δ were used to indicating the standardized difference between mean and median values, respectively, of selected cardiometabolic biomarkers after controlling for the presence of MetS and IR as defined by the SPISE cutpoints. Values of *d* of 0.20, 0.50 and 0.80 denote small, medium and large differences between means^17^, whereas, the absolute value of δ can be considered small around 0.15, medium around 0.33, and large around 0.47^18^.

## Results

**Figure 1:**
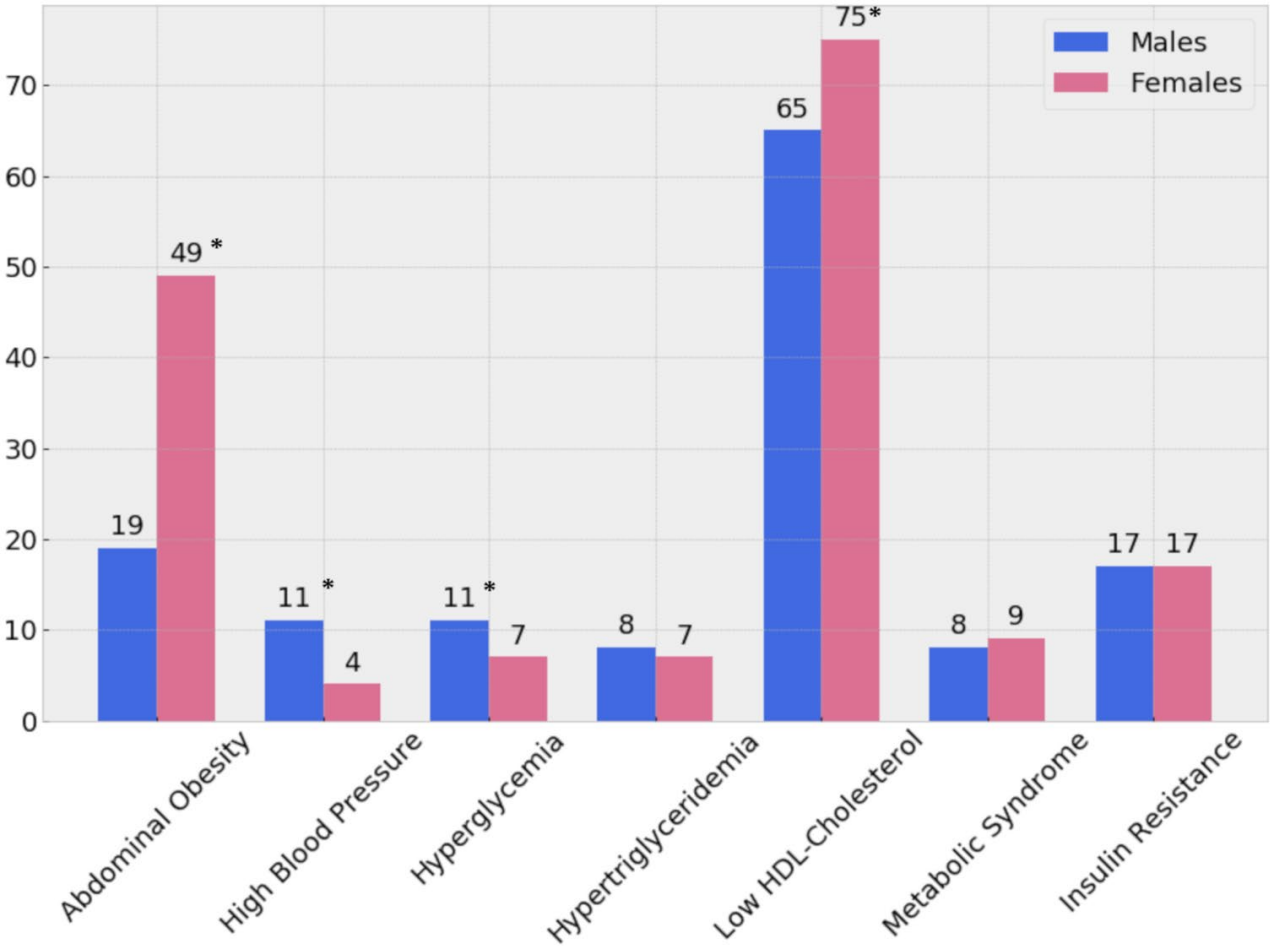
Cardiometabolic risk at 16.8y in the Santiago Longitudinal Study (n = 678) (prevalence rate, %). Metabolic Syndrome and its components diagnosed according to the AHA/NHLBI/IDF joint standard for people 16y and older. Insulin resistance diagnosed with HOMA-IR values ≥ 2.6. χ^2^ Pearson: *Statistical significance.

Anthropometric and cardiometabolic characteristics are presented in Table 1. A total of n = 678 participants (52% males) were evaluated. Participants' mean age was 16.8y (0.3 SD), and they all had completed pubertal development (Tanner 5). Males had significantly higher values of systolic blood pressure (SBP), diastolic blood pressure (DBP), and fasting glucose than females. Also, they had lower insulin, adiponectin, hs-CRP, and HDL-chol compared to females. No sex differences were found in the prevalence of obesity, IR, and MetS (see also Fig. 1).

**Table 1:** Anthropometric characteristics and Metabolic Syndrome related biomarkers in male and female post-pubertal adolescents (*n* = 678).

Variables	Males (n = 356)		Females (n = 322)		*P* value
	Mean or median	(SD) or [IQR]	Mean or median	(SD) or [IQR]	
Age (years)	16.8	(0.2)	16.8	(0.3)	NS
Body-Mass Index (z score)	0.57	(1.2)	0.74	(1.1)	NS
Obesity (BMI ≥ 30)	13.5%	48	14.6%	47	NS^ǂ^
Waist circumference (cm)	81.2	(10.8)	81.3	(11.5)	NS
Waist-to-height ratio	0.47	(0.1)	0.51	(0.1)	< 0.001
Waist-to-hip ratio	0.86	(0.1)	0.84	(0.1)	< 0.001
Systolic Blood Pressure (mm Hg)	114.7	(10.3)	108.2	(9.2)	< 0.001
Diastolic Blood Pressure (mm Hg)	71.0	(7.1)	67.2	(6.7)	< 0.001
Fasting glucose (mg/dl)	90.6	(9.5)	86.5	(8.9)	< 0.001
Fasting insulin (uUI/dl)	6.1	[4.1]	7.1	[4.9]	0.016^§^
HOMA-IR	1.4	[1.1]	1.5	[1.0]	NS^§^
SPISE	7.7	(2.1)	7.5	(2.1)	NS
Total cholesterol (mg/dl)	147.4	(24.2)	156.8	(26.9)	< 0.001
HDL cholesterol (mg/dl)	38.0	(9.6)	42.6	(10.5)	< 0.001
Triglycerides (mg/dl)	71.6	[45.3]	76.1	[44.7]	NS^§^
TG/HDL-chol ratio	2.05	[1.7]	1.84	[1.2]	0.022^§^
LDL cholesterol (mg/dl)	90.8	[28.4]	94.2	[29.9]	0.01^§^
hs_C-Reactive Protein (mg/dl) (n = 651)	0.36	[0.9]	0.45	[1.0]	0.047^§^
Adiponectin (µg/ml)	9.5	[5.8]	11.5	[6.6]	< 0.001^§^
Insulin Resistance (%)	16.8%	60	17.4%	56	NS^ǂ^
Metabolic Syndrome^a^ (%)	7.6%	27	9.0%	29	NS^ǂ^
Having 1 cardiometabolic risk factor^a^ (%)	46.1%	164	43.0%	138	NS^ǂ^
Having 2 cardiometabolic risk factor^a^ (%)	16.3%	58	35.1%	113	< 0.001

Values are Mean ± SD, Median(IQR) and relative frequencies (%). Two-tailed Student’s *t* test for independent samples, except as indicated. ^§^Wilcoxson rank-sum test. ^ǂ^Pearson’s χ2 test for statistical independence. Participants with hs_C-Reactive Protein > 9 were excluded from the analysis (n = 27). ^a^MetS and cardiometabolic risk assessed with the AHANHLBI/IDF joint standard for people 16y and older.

In males, the optimal cutoff for MetS diagnosis was 5.0. At this point, the sensitivity and specificity of SPISE as a diagnostic tool were 97% and 82% (Table 2). AUC denotes a very good diagnostic performance. The optimal cutoff for IR prediction in males was 5.9. At this point, the sensitivity and specificity of SPISE were 71% and 83%. AUC suggests a good diagnostic performance. In females, a SPISE of 6.0 had the highest sensitivity and specificity for MetS prediction. AUC indicates a very good diagnostic performance. In this group, a SPISE of 6.4 was the optimal cutoff for IR prediction; however, the sensitivity, specificity, and AUC suggest a fair-to-good diagnostic performance. While MetS and IR are knowingly more prevalent among adolescents with overweight and obesity, it may occur in normal-weight subjects with reduced muscle tissue. In our sample, 61.4% (n = 417) were normal-weight adolescents, of whom 10% had MetS and/or IR, and 50% had at least two cardiometabolic risk factors. Performance of SPISE for MetS diagnosis in the normal-weight group was good (AUC = 85.9%). However, performance for IR diagnosis was fair (AUC = 74.9%) (data not shown).

**Table 2:** Optimal cutoff values of SPISE to predict Metabolic Syndrome and Insulin Resistance in male and female post-pubertal adolescents.

	Cutoff	Sensitivity (%)	Specificity (%)	Correctly classified (%)	LR +	AUC
**Males (n = 356)**						
Metabolic syndrome	5.0	97.3	81.6	82.6	5.2	0.97
Insulin resistance	5.9	70.5	83.6	80.9	4.2	0.80
**Females (n = 322)**						
Metabolic syndrome	6.0	89.7	74.4	76.0	3.5	0.90
Insulin resistance	6.4	61.4	75.1	72.7	2.5	0.75

LR: Likelihood Ratio. AUC: Area under curve. MetS diagnosed with the AHANHLBI/IDF joint standard for people 16y and older. Insulin resistance diagnosed with HOMA-IR values ≥ 2.6

Because diagnosis is a procedure to guide the clinical choice to the best course of action, from a clinical viewpoint, the relevant question has to do with the chance that the condition will be present when a positive test result is obtained^19^. There lies the importance of making a difference between pre- and post-test probabilities of disease. The pretest probability is the proportion of people in the population at risk who have the disease at a specific time or time interval (e.g., the point prevalence or the period's prevalence) before the test is performed. The post-test probability denotes the proportion of individuals testing positive who genuinely have the disease. It is similar to the positive predictive value, but apart from the test performance also includes a patient-based probability of having the disease. Table 3 contains the probabilities of the presence of the MetS and IR before and after using the SPISE. In our male participants, the pretest probability of having the MetS was 7.7% before the SPISE. After the SPISE, for those with values below the optimal cutoff (positive test), the chances of having the disease increased to 30%. In the same group, the pretest probability of having IR was 16.8% before the SPISE. After the SPISE, for those having values below the optimal cutoff, the chances of having the disease increased to 45%. The same pattern was found in females, for whom the probability of having these conditions increased notably in the group with SPISE values below the optimal cutoff points.

**Table 3:** Pre- and post-test probability of the presence of Metabolic Syndrome and Insulin Resistance in male and female post-pubertal adolescents (*n* = 678).

	SPISE cutoff	Pre-test probability or prevalence rate	Post-test probability (having the test positive) (95% CI)	Post-test probability (having the test negative) (95% CI)
**Males (n = 356)**				
Metabolic syndrome	5.0	7.7%	31.0% (21.0–40.2)	1.3% (0.5–3.3)
Insulin resistance	5.9	16.8%	45.2% (34.7–55.2)	6.4% (4.3–10.7)
**Females (n = 322)**				
Metabolic syndrome	6.0	9.0%	26.1% (20.2–29.9)	1.4% (1.1–5.3)
Insulin resistance	6.4	17.4%	34.7% (24.1–42.9)	9.5% (6.3–12.0)

MetS diagnosed with the AHANHLBI/IDF joint standard for people 16y and older. Insulin resistance diagnosed with HOMA-IR values ≥ 2.6

**Figure 2:**
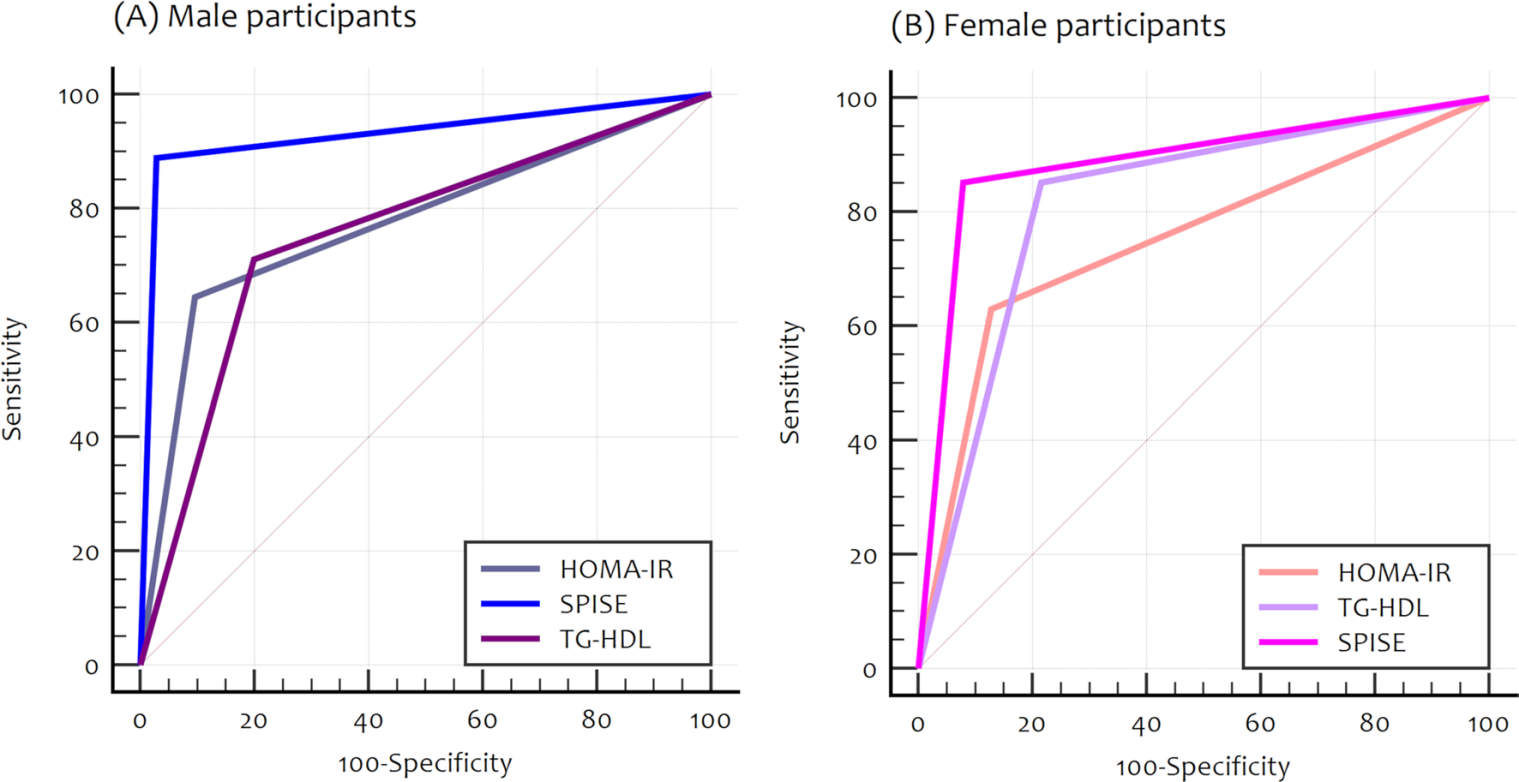
Pairwise comparison of ROC Curves for Metabolic Syndrome diagnosis: SPISE, HOMA-IR and TG-HDL ratio. A test with perfect discrimination has a ROC plot that passes through the upper left corner, an indication of 100% sensitivity and 100% specificity. A ROC plot closer to the upper left corner denotes greater accuracy of the test. Metabolic Syndrome and its components diagnosed with the AHA/NHLBI/IDF standard for people 16y and older.

Next, we compared the diagnostic performance of SPISE, HOMA-IR, and TG-HDL ratio for MetS prediction in males and females (Fig. 2 and Table S1). In males, although HOMA had a good performance in the prediction of MetS, with an AUC of 80%, we found that SPISE was superior since the AUC was ≥ 90%, and the difference in the areas was statistically significant (*P* < 0.0001). A comparison of SPISE vs. TG-HDL for MetS diagnosis in males showed that SPISE had a significantly larger AUC (95% vs. 80%; *P* < 0.0001), suggesting a better diagnostic performance. In females, both HOMA and TG-HDL had significantly smaller AUCs compared with SPISE, which confirms that SPISE outperforms both in the diagnosis of MetS.

**Figure 3:**
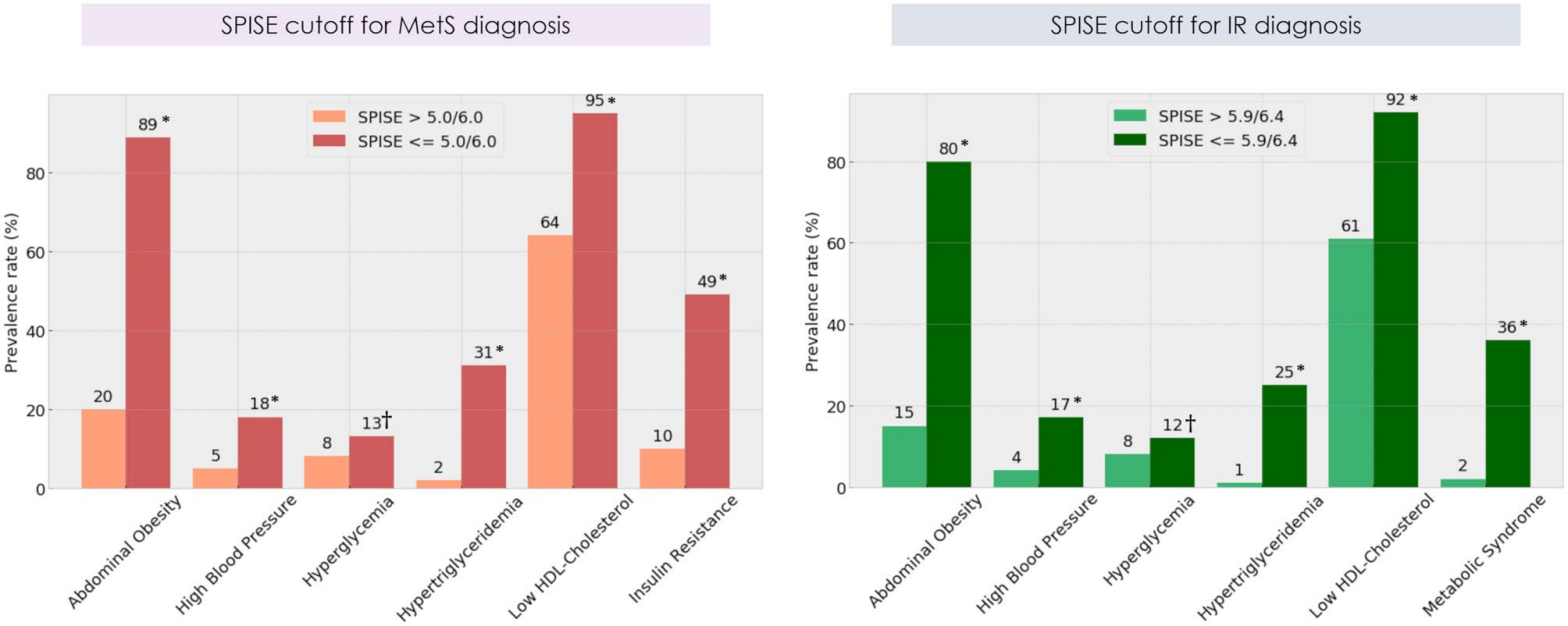
Prevalence of cardiometabolic risk in the sample according to optimal SPISE cutoffs for MetS and IR diagnosis. Metabolic Syndrome and its components diagnosed with the AHA/NHLBI/IDF standard for people 16y and older. Insulin Resistance: HOMA-IR ≥ 2.6. Pearson’s χ^2^ test: *Statistical significance. †Trend towards statistical significance. SPISE for MetS diagnosis: ≤ 5.0 and ≤ 6.0 in males and females, respectively. SPISE for IR diagnosis: ≤ 5.9 and ≤ 6.4 in males and females, respectively.

Last, we checked whether our SPISE cutpoints related to higher biological risk in the group having a SPISE below the cutpoints for MetS and IR diagnosis. A higher prevalence of hypertriglyceridemia and low HDL was found in the group having the test positive. However, we also found a higher prevalence of abdominal obesity and high blood pressure and a trend towards a higher prevalence of hyperglycemia, whose biomarkers are not included in the SPISE algorithm (Fig. 3). Particularly, the prevalence of abdominal obesity was 4.5 and 5.3 times higher in adolescents having SPISE below the cut points for MetS and IR diagnosis, respectively, compared to those with SPISE values above those thresholds. Likewise, the prevalence of hypertension was 3.6 and 4.3 times higher in the group testing positive for MetS and IR, respectively, using SPISE. Table 4 contains the cardiometabolic profile of participants after controlling for sex and MetS and IR presence, according to SPISE cutoffs. In males and females, participants having a SPISE below the cutoff for MetS prediction had significantly higher values of WC, SBP, DBP, glycemia, insulin, HOMA-IR, TChol, LDL-chol, TG, TG/HDL-chol ratio, and hs-CRP, and significantly lower values of HDL-chol compared to males and females having the test negative. Notably, the effect size for difference was also large for biomarkers not included in the SPISE algorithm, such as WC, SBP, TChol, HOMA-IR, and insulin in both sexes, and LDL-chol in males. Likewise, males and females having SPISE values below the cutoff for IR prediction had an unhealthier cardiometabolic profile compared to peers with a test negative. Again, the effect size for the difference was large for biomarkers not included in the SPISE formula, such as WC, SBP, insulin, HOMA-IR, adiponectin in males, and WC, SBP and hs-CRP in females.

**Table 4:** Cardiometabolic profile according to optimal cutoff values of single point insulin sensitivity estimator (SPISE) for metabolic syndrome and IR diagnosis.

	Metabolic syndrome				Insulin resistance			
	SPISE > 5.0	SPISE ≤ 5.0	*t* or *z* statistic	ES for difference^†^	SPISE > 5.9	SPISE ≤ 5.9	*t* or *z* statistic	ES for difference
	(n = 307)	(n = 49)			(n = 269)	(n = 87)		
**Males (n = 356)**								
Waist circumference (cm)	78.1 ± 7.5	100.4 ± 10.1***	18.4	2.83	76.5 ± 6.2	95.5 ± 10.3***	20.7	2.55
Systolic Blood Pressure (mm Hg)	113.4 ± 10.3	122.5 ± 9.8***	5.93	0.91	112.6 ± 9.5	120.9 ± 10.4***	6.79	0.83
Diastolic Blood Pressure (mm Hg)	69.8 ± 6.7	75.0 ± 7.5***	4.91	0.75	69.6 ± 6.9	73.3 ± 7.0***	4.30	0.53
Fasting Glucose (mg/dl)	89.9 ± 8.5	94.7 ± 13.7**	3.33	0.49	90.2 ± 8.4	91.7 ± 12.5	1.28	0.16
Fasting Insulin (uUI/dl)	5.6 (4.0)	14.0 (8.3)***	8.30	0.73^**‡**^	5.4 (3.7)	11.2 (7.9) ***	9.37	0.67^**‡**^
HOMA-IR	1.27 (1.6)	3.27 (2.4)***	8.28	0.74^**‡**^	1.22 (0.9)	2.41 (2.12) ***	8.96	0.64^**‡**^
Total Cholesterol (mg/dl)	144.2 ± 21.7	167.5 ± 29.1**	6.63	1.01	144.0 ± 21.5	158.1 ± 28.6	4.88	0.60
HDL-cholesterol (mg/dl)	39.1 ± 9.9	30.7 ± 6.4***	5.68	0.87	40.2 ± 9.9	31.0 ± 6.1***	7.16	0.88
Triglycerides (mg/dl)	67.5 (36.6)	133.5 (101.9)***	8.94	0.80^**‡**^	63.9 (32.1)	115.5 (77.9) ***	9.71	0.69^**‡**^
TG/HDL ratio	1.80 (1.2)	4.15 (3.4)***	9.49	0.84^**‡**^	1.72 (1.0)	3.89 (2.7)	11.1	0.79^**‡**^
LDL-cholesterol (mg/dl)	88.9 (21.9)	106.3 (24.1) ***	4.77	0.82	88.5 (21.6)	101.5 (22.4)***	3.86	0.62^**‡**^
hs_C-Reactive Protein (mg/dl) (n = 341)	0.32 (0.7)	0.91 (1.9)***	4.26	0.40^**‡**^	0.3 (0.7)	0.7 (1.7)**	4.83	0.35^**‡**^
Adiponectin (µg/mL)	9.73 (5.6)	6.74 (4.7) ***	4.53	0.47^**‡**^	10.1 (6.0)	6.72 (4.5) ***	6.47	0.49^**‡**^

	Metabolic syndrome				Insulin resistance			
	SPISE > 6.0	SPISE ≤ 6.0	*t* or *z *statistic	ES for difference^†^	SPISE > 6.4	SPISE ≤ 6.4	*t* or *z *statistic	ES for difference
	(n = 243)	(n = 79)			(n = 223)	(n = 101)		
**Females (n = 322)**								
Waist circumference (cm)	76.9 ± 8.1	94.8 ± 11.0***	15.5	2.00	75.5 ± 7.4	93.5 ± 10.3***	17.5	2.09
Systolic Blood Pressure (mm Hg)	106.0 ± 8.3	115.2 ± 10.2***	7.96	1.03	105.7 ± 8.4	114.0 ± 9.9***	7.53	0.90
Diastolic Blood Pressure (mm Hg)	66.5 ± 6.5	70.3 ± 6.7***	4.48	0.58	66.0 ± 6.2	70.6 ± 6.7***	5.97	0.72
Fasting Glucose (mg/dl)	85.8 ± 8.7	88.4 ± 9.3*	2.21	0.29	85.7 ± 8.7	88.0 ± 9.2	2.11	0.25
Fasting Insulin (uUI/dl)	6.59 (3.9)	10.8 (8.8)***	6.99	0.52^**‡**^	6.5 (4.1)	9.0 (7.0) ***	6.42	0.45^**‡**^
HOMA-IR	1.39 (0.9)	2.27 (1.9)***	6.95	0.52^**‡**^	1.38 (0.9)	1.99 (1.6) ***	6.37	0.44^**‡**^
Total Cholesterol (mg/dl)	154.2 ± 27.4	163.1 ± 24.7*	2.38	0.31	154.0 ± 23.3	163.2 ± 32.8**	2.87	0.34
HDL-cholesterol (mg/dl)	44.5 ± 10.8	37.0 ± 8.4***	5.66	0.73	44.6 ± 11.0	38.4 ± 8.6***	4.99	0.60
Triglycerides (mg/dl)	71.6 (34.1)	103.3 (72.8)***	8.94	0.50^**‡**^	70.7 (33.6)	96.6 (71.1) ***	6.75	0.47^**‡**^
TG/HDL ratio	1.65 (0.9)	2.66 (2.2)	7.82	0.58	1.61 (0.9)	2.50 (2.1)	7.61	0.53^**‡**^
LDL-cholesterol (mg/dl)	94.9 (22.0)	102.0 (25.1)*	2.12	0.32	94.2 (21.8)	102.0 (26.8)*	2.45	0.33^**‡**^
hs_C-Reactive Protein (mg/dl) (n = 309)	0.34 (0.8)	1.16 (1.8)***	5.76	0.44^**‡**^	0.32 (0.7)	1.16 (1.9) ***	6.56	0.47^**‡**^
Adiponectin (µg/mL)	12.2 (6.9)	9.52 (6.3) ***	4.32	0.33^**‡**^	12.3 (6.8)	9.62 (6.6) ***	4.10	0.30^**‡**^

MetS diagnosed with the AHANHLBI/IDF joint standard for people 16y and older. Insulin resistance diagnosed with HOMA-IR values ≥ 2.6. Values are Mean ± SD and Median(IQR). Student’s *t* test. ^2^ Wilcoxson rank-sum test. *Significant at *P* < 0.05. **Significant a *P* < 0.01. ***Significant at *P* < 0.001. ^†^Cohen’s *d* statistic, except as indicated. ^‡^Cliff’s *δ* statistic for non-normal distributions. Values of *d* of 0.20, 0.50 and 0.80 denote small, medium and large differences. Values of *δ* are considered small around 0.15, medium around 0.33, and large around 0.47. Participants with hs_C-Reactive Protein > 9 were excluded from the analysis (n = 27).

## Discussion

### Main findings

We found that IR-related cardiometabolic risk in post-pubertal adolescents can be estimated using the SPISE, a new, low-cost, simple to estimate index. In males and females, SPISE had a very good and good diagnostic performance for predicting MetS. In both sexes, SPISE showed a significantly better ROC curve than HOMA-IR for MetS diagnosis. Although SPISE was an accurate diagnostic tool for IR prediction in males, this was not always the case for females. Still, diagnostic performance in females was fair-to-good at an AUC of 75%.

Few studies have conducted validity assessments of SPISE for the prediction of IR-related cardiometabolic disorders. They have done so using different populations and study designs. The index was developed based on the TG, HDL-chol, and BMI in two European cohorts of individuals with obesity^8^: the β-Cell Function in Juvenile Diabetes and Obesity (Beta JUDO) study cohort (n = 29; mean age 15y), and the Relationship between Insulin Sensitivity and Cardiovascular Disease (RISC) study cohort (n = 1,260; mean age 44y). In both samples, oral-glucose-tolerance tests and hyperinsulinemic-euglycemic clamp were used to estimate insulin sensitivity and calculation of insulin sensitivity indices. Mathematical modeling was applied, including BMI, fasting TG, and HDL-chol and compared to the clamp M-values using ROC analysis. In both youth and adults with obesity, a SPISE of 6.61 was the optimal cutpoint for diagnosing IR (M-values of < 4.7 mg/ kg/min). SPISE slightly underperformed the Matsuda ISI in the prediction of IR, performed equally to the QUICKI and HOMA-IR, and outperformed the TG/HDL-chol ratio. The SPISE accuracy for the prediction of cardiometabolic risk was later tested among adults from Northern India^20^. In a community-based cross-sectional study including n = 229 MetS cases (mean age 46.9y) and 248 controls (mean age 38.4y), Dudi et al. found that a SPISE of 5.82 had a good predictive ability to discriminate the MetS. The mean value of SPISE was found to be significantly lower in MetS patients than controls (5.35 vs. 7.45).

Our results show that SPISE performed significantly better than HOMA in the prediction of clustered cardiometabolic risk. It seems that SPISE characterizes well the role of proatherogenic conditions (e.g., inflammation and abnormal lipoprotein metabolism) in obesity-related cardiometabolic disorders. Inflammation plays an important role in the development of IR through different cytokines and molecular pathways^21^. In our sample, participants with a SPISE below the cutpoints for MetS diagnosis had remarkably higher levels of hs-CRP and lower adiponectin than participants with SPISE values above the cutpoint. The differences were moderate for hs-CRP in both sexes and moderate and large for adiponectin in females and males. Obesity, particularly intra-abdominal obesity, relates to chronic low-grade systemic inflammation and low adiponectin, an important predictor of cardiovascular risk. In our sample, the prevalence of abdominal obesity was 4.5 times higher in participants with SPISE values below the cut-off point for MetS prediction. Similarly, mean waist circumference was much larger in males and females having a reduced SPISE. A similar deterioration pattern was seen for TChol, HDL-chol, and TG in both sexes and LDL-chol in males. It has been found that a dysfunctional insulin signaling in peripheral tissues (e.g., white adipose tissue) in the early stages of IR leads to an abnormal lipid metabolism that results in a pro-atherogenic phenotype^22,23^. A study conducted in Sweden obtained results that might be consistent with ours, although in a sample of older adults. In 71-year-olds from the Uppsala Longitudinal Study of Adult Men, Cederholm and Zethelius found that the SPISE performed as well as the QUICKI, log HOMA-IR and revised QUICKI as a predictor for future risk of fatal and non-fatal coronary heart disease^24^.

A comparison of SPISE with the TG/HDL ratio for MetS diagnosis in this sample of post-pubertal adolescents showed that SPISE significantly outperformed the TG/HDL ratio in the screening of clustered cardiometabolic risk. Differences in terms of area under the ROC curve were 15 percentage points in males and eight percentage points in females. While the diagnostic performance of MetS using the TG/HDL-chol ratio may be considered good (based on AUCs of 0.80 and 0.82 in males and females, respectively), the diagnostic performance of SPISE increased to 95% in males and 90% in females, which is considered to be excellent and very good, respectively. Therefore, the inclusion of BMI in the atherogenic index and some mathematical modeling substantially improved its screening capability. This procedure can be easily implemented in an algorithm using spreadsheets or other statistical software that operates on data entered in table cells. This requires minimal computational effort while retaining reasonable accuracy and allows estimation and tracking of the SPISE at the individual level. Continuous monitoring of SPISE may serve for the rapid identification of clinically relevant changes and help guide treatment. Because ethnic differences in the TG/HDL-C threshold were identified in several studies^25–27^, future research should explore the extent to which this holds for the SPISE.

A fourth significant finding relates to the fact that SPISE had greater diagnostic accuracy in males compared to females. This is in line with evidence describing a sexual dimorphism in incidence, age of onset, and progression of most cardiometabolic diseases, with males generally showing less beneficial profiles^28–31^. In our sample, males had a more adverse cardiometabolic profile than females that may put them at higher risk of IR and MetS. It is known that sex influences body fat distribution, ectopic fat accumulation, insulin signaling, glucose homeostasis, and lipid metabolism. Thus, the challenge is to consider those differences in both clinical practice and epidemiological screenings. To the best of our knowledge, this is the first validity assessment of SPISE considering sex-related differences in this biomarker's efficacy.

### Main implications

In adolescents, SPISE might be a promising tool for estimating IR-related cardiometabolic risk in both clinical and epidemiological settings. Although IR is standard in children and adolescents with obesity and relates to a higher risk of major cardiometabolic disorders^1–3^, IR prevalence in these groups is not well established^5^. According to Levy-Marchal et al., screening tests based on fasting insulin measurements have not been able to provide accurate, reliable, reproducible, and easily applicable measurement procedures^5^. Even in clinical practice, insulin is not advised to measure insulin sensitivity in the pediatric population, and the same holds for surrogate methods such as HOMA and QUICKI^5,32^. Hence, there is a need to have available screening programs to assess insulin sensitivity without measuring insulin levels. SPISE is based on BMI and routine lipids, which are much cheaper to obtain, more reliable than insulin, and require a single blood sample.

Second, SPISE opens up the possibility to identify adolescents at risk of IR-related cardiometabolic disorders in large groups. Because of the vast number of youths having or are at risk of obesity and because IR may occur as part of the physiological changes in puberty^1,2^, early detection of impaired insulin sensitivity in adolescents is pivotal to designing targeted preventive actions. Population health surveys usually have measurements of body-mass index and lipid profile. Hence, SPISE could be used to screen IR-related cardiometabolic risk in ethnic groups that are more insulin resistant than white Europeans, regardless of body-mass index, total fat mass, and visceral adiposity. It is the case of Hispanics, African Americans, and East Asians^33–35^, or people living in countries undergoing rapid industrialization significant increases in dietary fat and sugar intake and persistent declines in physical activity levels. Furthermore, these groups might have inherited the so-called thrifty phenotype^36^. This is an adaptive mechanism engineered to protect the brain, at the expense of other tissues such as the pancreas, in the face of food scarcity. In the long-term, however, the mechanism predisposes to increased risk for cardiometabolic abnormalities, manifested first as inadequate glycemic control and later as type-2 diabetes and its complications^36^.

Last, because sex has an impact on several determinants of insulin sensitivity it is important to consider sex differences in glucose metabolism and insulin action and, thus, sex-specific standards when measuring IR-related cardiometabolic risk are also needed.

### Strengths and limitations

This study has some limitations. Because our sample was comprised of post-pubertal adolescents from low- to middle SES between a narrow age-range: 16 to 17y, our findings cannot be generalized to the overall population of Chilean adolescents. Secondly, although we used the IDF/AHA/NHLB criteria for MetS diagnosis, which is the consensus of several major organizations to unify diagnosis criteria of MetS in individuals ≥ 16y, IR in our sample was diagnosed using the HOMA-IR, which is derived from fasting insulin and glucose concentrations, instead of using the glucose clamp. However, because the glucose clamp is an invasive procedure, it is not easy to use in healthy individuals. Third, the cross-sectional nature of the study constraints the ability to conclude on the temporality of these associations. Future studies should longitudinally explore this indicator's performance in predicting the risk of cardiometabolic disorders later in life.

On the other hand, our study has several strengths. According to population-based surveys and national studies, the prevalence of obesity and cardiometabolic risk is much higher in adolescents of low- to middle SES. They are more exposed to risk factors that lead to obesity, IR, and MetS than high-SES adolescents^4,10,12,24,28,37^. Second, we provide evidence of a biomarker that allows good early discrimination of adolescents with IR-related cardiometabolic risk, using a low-cost, easy-to-estimate indicator based on biological risk. Hence, it might be potentially useful in both clinical and population settings. Third, we found sex differences in this biomarker's effectiveness to identify adolescents at higher cardiometabolic risk. The sexual dimorphism has not been described in previous validity assessments of the SPISE. Also, we estimated post-test probabilities. While post-test probabilities may be quite useful in everyday clinical work, they are often roughly estimated or even guessed. When they are calculated, clinical decision-making may rely on pure quantitative criteria, allowing appropriate and comprehensive use of results from screening tests. If more sophisticated or expensive screening methods are needed, or resources for interventions are scarce, the post-test probabilities allow focusing on those at higher biological risk. Second, post-test probabilities help to determine which test is best for the patient, in terms of costs and safety, using the most economical and safest option by which an acceptable post-test probability can be achieved. Third, it is possible to determine whether the probability of a positive diagnosis has risen (i.e., the post-test probability has increased) or fallen (i.e., post-test probability has decreased). Another strength of the study has to do with the ethnic background of participants. Our sample consists of Hispanic adolescents, and according to the evidence, this is a group less insulin sensitive than Caucasian adolescents^38–40^.

## Supplementary information

Supplementary file1

## Data Availability

The datasets generated during and/or analyzed during the current study are available from the corresponding author on reasonable request.
