# Supplementary material for: Validity assessment of the single-point insulin sensitivity estimator (spise) for diagnosis of cardiometabolic risk in post-pubertal hispanic adolescents
Source: Sci Rep. 2020 Sep 1;10:14399. doi: 10.1038/s41598-020-71074-y (PMC7462984; doi:10.1038/s41598-020-71074-y)
Supplement: Supplementary file 1 — Supplementary file1 [file 41598_2020_71074_MOESM1_ESM.pdf]

SUPPLEMENTARY MATERIAL

VALIDITY ASSESSMENT OF THE SINGLE-POINT INSULIN SENSITIVITY ESTIMATOR (SPISE) FOR DIAGNOSIS OF CARDIOMETABOLIC RISK IN POST-PUBERTAL HISPANIC ADOLESCENTS

Authors: Paulina Correa[1], Estela Blanco[2], Sheila Gahagan[2], Raquel Burrows[1*]

Affiliation: [1]Instituto de Nutrición y Tecnología de Alimentos, Universidad de Chile (UCH), Chile. [2]Child Development and Community Health, University of California-San Diego (UCSD), USA.

Corresponding author: Prof. Raquel Burrows. Instituto de Nutrición y Tecnología de los Alimentos. Universidad de Chile. Avda. El Líbano 5524, Macul. CP: 7830490. Santiago de Chile, Chile. Tel.+56 2 29781492. Fax: +56 2 22214030. E-mail: rburrows@inta.uchile.cl.

Table S1 Area under curve pairwise comparison for MetS diagnosis: SPISE, HOMA-IR and TG-HDL ratio

Male post-pubertal adolescents (n=356)					
SPISE vs. HOMA-IR		HOMA-IR vs. TG-HDL ratio		SPISE vs. TG-HDL ratio	
AUC_{SPISE}	0.968	$AUC_{HOMA\text{-}IR}$	0.816	AUC_{SPISE}	0.968
$AUC_{HOMA\text{-}IR}$	0.816	$AUC_{TG\text{-}HDL}$	0.800	$AUC_{TG\text{-}HDL}$	0.800
$AUC_{SPISE} – AUC_{HOMA\text{-}IR}$	0.152	$AUC_{HOMA\text{-}IR} – AUC_{TG\text{-}HDL}$	0.016	$AUC_{SPISE} – AUC_{HOMA\text{-}IR}$	0.168
Standard Error	0.040	Standard Error	0.048	Standard Error	0.031
95% CI	0.077 - 0.235	95% CI	-0.076 - 0.112	95% CI	0.114 - 0.234
z statistic	3.862	z statistic	0.377	z statistic	5.665
Significance level	*P* < 0.001	Significance level	*P* = 0.706	Significance level	*P* < 0.001

Female post-pubertal adolescents (n=322)					
SPISE vs. HOMA-IR		HOMA-IR vs. TG-HDL ratio		SPISE vs. TG-HDL ratio	
AUC_{SPISE}	0.896	$AUC_{HOMA\text{-}IR}$	0.751	AUC_{SPISE}	0.896
$AUC_{HOMA\text{-}IR}$	0.751	$AUC_{TG\text{-}HDL}$	0.818	$AUC_{TG\text{-}HDL}$	0.818
$AUC_{SPISE} – AUC_{HOMA\text{-}IR}$	0.145	$AUC_{HOMA\text{-}IR} – AUC_{TG\text{-}HDL}$	0.067	$AUC_{SPISE} – AUC_{HOMA\text{-}IR}$	0.078
Standard Error	0.062	Standard Error	0.068	Standard Error	0.029
95% CI	0.013 - 0.257	95% CI	-0.067 – 0.201	95% CI	0.012 to 0.125
z statistic	2.175	z statistic	0.980	z statistic	2.367
Significance level	*P* = 0.029	Significance level	*P* = 0.327	Significance level	*P* = 0.018

AUC: Area under Curve
